# Local Anæsthesics

**Published:** 1893-03

**Authors:** N. S. Hoff


### Local Anæsthetics.

BY N. S. HOFF, D.D.S.

Read before the Detroit Dental Society, February 13th, 1893.

Much confusion exists in our habit of referring to the various classes of agents used to relieve or prevent pain. Without thinking, we often speak or think of narcotics, anodynes and anæsthetics as synonymous terms. It may make our subject more lucid and profitable should we indulge in a brief definition of these terms.

Narcotics are drugs which will induce sleep more or less profound and similar to the natural process. Primarily these agents stimulate the circulatory system and carry the drug, by the increased circulation, to the ultimate cell tissue of the body and there produce a sedative effect upon them; the nerve centers are abolished and functional activity of the various specialized tissues and organs of the body is interrupted. Narcotics do not usually select a special organ, but affect the entire system sooner or later in their career. Belladonna, however, seems to be an exception, in that it produces the usual symptoms of narcotics; at the same time there is a certain amount of excitement or delirium, manifested in the desire to speak or move about. The brain and cord are affected, and through them all tissues of the body by narcotics.

Anodynes are remedies which relieve pain. This term is not applied to such agents as prevent pain, for instance in a surgical operation, but they are usually applied to relieve pain in or resulting from pathological conditions; hence they may be such agents as will control circulation in painful inflammatory conditions, because of a localized action, as for instance an astringent; or they may allay pain by a profound action upon the central nervous system and control local or general pain, as exemplified in internal application of opium. Briefly and practically anodynes are medicines, and for pathological conditions.

Anæsthetics are agents which destroy sensation. Anæsthetics produce insensibility in much the same way that narcotics and anodynes do, namely by affecting the nervous system, but therapeutically they are used for entirely different purposes, and hence, although an anæsthetic may be a narcotic or anodyne to a certain extent, yet anæsthetics are entitled to a peculiar classification in therapeutics because they are not remedial agents, but expedients. We use anæsthetics to prevent pain when operating upon tissues endowed with normal sensibility, and their effects are transitory, because of their volatility, while narcotics are in a sense curative and their influence enduring. Anodynes do not in average doses affect the reflexes, while anæsthetics abolish reflex.

Anæsthetics are divided into two classes, general and local. The general are those which are administered to the extent of affecting the entire system, and produce absence of sensation in all parts by action upon the control nerve centers. It is questionable whether they affect first the sensory fibers or not; but this is immaterial since it is evident that the nerve centers themselves are profoundly affected.

The local anæsthetics in dental therapeutics are again divided into local anæsthetics and obtundents. The local anæsthetics are those agencies made use of to produce insensibility in the soft tissues involved in dental surgery, while obtundents are those agents which are used to render operations upon living dentine painless. The time at my disposal this evening will not permit any discussion of the various obtundent agencies or the manner in which they operate, and as I presume the program committee intended a consideration only of the agents used in securing painless operations in the soft structure, I shall confine my consideration entirely to this class.

There are two classes of local anæsthetics based on the manner of application and effects. One of these classes includes the natural resources, such as electricity and cold; the other embraces the various drugs or chemicals applied directly to the tissues by epidermic methods or by hypodermic injections.

Of electricity we probably do not, at present, obtain as favorable results, and it consequently is not generally recognized as a practical local anæsthetic, although it has many advocates and has for many years claimed no little attention from the profession. Medical electro-therapeutics is only in its infancy, and local anæsthesia from this source is one of the hopeful signs of the times. When we are fortunate enough to have dental electricians we will hope to reach favorable results.

The methods of applying and using this agency are so familiar to you that I shall refer to it as briefly as possible. It has been applied extensively in extracting teeth, by attaching to one electrode of an ordinary galvanic battery the forceps, the handles of which are covered with rubber. While the patient holds the other electrode in the hand, the operator applies the forceps to the tooth and turns on the current at the proper strength.

In the hands of some dentists this has apparently been a satisfactory procedure, but I think it is not generally used, probably because of the shock to the patient and the inconvenience of the appliances.

Abolishing sensation by means of cold applications has met with more favor and is accomplished most easily by the evaporation of volatile agents upon the tissues involved. For a long time sulphuric ether, applied in the form of a spray, was used in preference to the general anæsthetics. Its application is made by means of an ordinary spray apparatus, the spray nozzle of which is bifurcated so as to allow one opening for throwing the spray on to the gum on each side of the tooth at the same time. The mouth is then protected with napkins, so that only the parts to be operated upon are exposed and the spray applied continuously until the gums assume a blanched condition. The operation can then be performed with almost no pain. Care should be exercised so that the blanching is not produced too suddenly and on the surface only, otherwise the result will be unsatisfactory because the normal temperature of the deeper structures has not been sufficiently reduced to produce complete anæsthesia.

While this process is undoubtedly a successful one in attaining a definite result, it has its drawbacks. There is danger of destroying the life of the tissues by the excessive low temperature produced, resulting in necrosis of the gum and alveolus. The application is not conveniently made, is more or less disagreeable to the patient, and makes an unpleasant odor in the office. These considerations have undoubtedly brought about the disuse of the process. More recently chloride of methyl, chloride of ethyl and nitrous oxide have been advocated for this same method. While the apparatus for their application is somewhat simplified with these agents the results obtained are the same, and the objections above noted are identical. These methods will not likely ever become popular, but they may be used advantageously where other affective means are inapplicable, because of idiosyncrasies of patients in regard to general anæsthetics or drugs used in other ways.

Of chemical agents a great deal of use has been made of such as would by application to the mucous membrane be absorbed and produce sufficient insensibility as to permit painless operations. Our time and space will not permit of even a reference to the numerous agents of this class that have been recommended. All narcotic and anodyne drugs and some stimulating astringents have found favor. It is doubtless due to this experimentation that we are now able to accomplish desirable anæsthesia by our present approved methods. The nature of the gum tissue is such that these drugs are absorbed with great difficulty, and therefore prompt anæsthesia can not be obtained by their use. The difficulty experienced in producing satisfactory effects with the most potent drugs by absorption, led to hypodermic injections with more satisfactory results—so much so that this method has become very popular, because of prompt and profound impressions that can be made with properly selected drugs.

The discovery of cocaine marks an important era in the field of dental operative surgery, as it is now possible to secure absolutely painless operations with a minimum of danger and little or no expensive or unwieldly apparatus. It is not possible at this time to discuss this phase of local anæsthesia without giving largely the history and action of cocaine, since it forms the basis of nearly all preparations used in local anæsthesia. I shall not attempt a complete history of this drug, but only call your attention to its practical application in this connection.

While it is true, there is no agent which will secure for us such prompt and effective results, there is also no drug capable, if carelessly or ignorantly used, of doing so much actual harm. In practical use we find the preparation known as the hydrochlorate is to be preferred. This is obtained in its best condition in the form of crystals, and as a convenience in one half grain capsules, or compressed tablets. Two per cent. solutions should be made with carbolated distilled water, a two per cent. solution. Cocaine alone is best used in a two per cent. solution for local anæsthesia. The addition of carbolic acid to the distilled water, helps to render the solution antiseptic and also limits the absorption of the cocaine into the general circulation, thereby localizing its action. If higher percentage solutions are used, some agents which will counteract the paralyzing effect of the cocaine on the heart and respiration should be added. The most effective agent of this kind is the sulphate of atropine. This is a valuable addition to the formula not only for its antagonistic effect but it also increases the local effect by paralyzing the nerve endings in the tissues involved.

In order therefore to construct a formula which will meet the demands of local anæsthesia and be safe to use, four elements at least are necessary, viz: the basis, the adjuvant, the corrective, the diluent. And as an illustration we will compound the following formula making a two per cent. solution of cocaine.

| | | |
|---|---|---|
| R. | Cocaine Hydrochlorate, grains | x—Basis. |
| | Sulphate of Atropine, grains | 1-10—Corrective. |
| | Carbolic acid, 95 per cent. solution, gtts | viij—Adjuvant. |
| | Distilled Water | ℥j—Diluent. |

Dissolve carbolic acid and atropine in the water and to every twenty five drops add one half grain of cocaine. When wanted for use, other drugs may be added to this formula to increase or intensify the effect of the cocaine, but in my judgment chloral, camphor and aconite which are usually employed do not materially increase the power of the formula, while it is quite certain that they do cause an excessive irritation that is somewhat difficult to control. Chloral is especially apt to cause this irritation, and in some cases it will produce excessive swelling or even sloughing of the gums. To increase the power of the formula I should prefer rather to increase the amount of cocaine up to a three or four per cent solution. The amount of this formula that can be safely used at one time can be determined by remembering that one half grain of cocaine is a safe dose for a hypodermic injection and that $\frac{1}{200}$ to $\frac{1}{60}$ of a grain of atropine can be safely injected. And since the atropine and cocaine neutralize each other in their physiological actions, a dose of each given at the same time must be safe. The carbolic acid in the quantity indicated is not at all dangerous and consequently we would be justified in using hypodermically twenty five drops of the above solution. But practically it will not generally be necessary to use more than ten or fifteen drops, unless a great many teeth are to be extracted. This formula is selected only as a basis or a study as it were, but it will be found useful in its present form. It is hoped, however, that this subject will be investigated both clinically and scientifically.

A few words as to the use of the syringe. The syringe should be in perfect order, and means for sterilizing the needle should certainly be used. Before introducing the needle into the gum it should be dipped into a strong solution of carbolic acid, and then washed in a five per cent. solution of the same, which should not be wiped off, but if a drop remains on the point when it touches the gum, it will paralyze the tissue so that the needle will not hurt when inserted. Insert the needle just below the margin of the gum and press gently and slowly into the deeper structures, and then slowly force the cocaine solution into the tissues, allowing time for its absorption. Remove needle and insert on the opposite side of the tooth, and repeat the process as often as necessary, depending on the extent of the operation. Each time after removing the needle press the finger firmly over the opening left by the needle to prevent escape of the cocaine. Allow two to five minutes for complete effects and operate.

To provide for any emergency such as fainting or collapse from the anæsthetic or shock, restorative agents should always be near at hand. Spirits of ammonia: Take a wide-mouth, tooth-powder bottle and place in the bottom a piece of absorbent cotton and saturate or dampen with ammonia; cork with a rubber stopper and keep in the medicine chest; it will come handy some time. Also keep some nitrite of amyl in little glass flasks or "tears" as they are called. About three drops in a little glass globule. In case of profound collapse break one of these tears in a handkerchief or small napkin and allow patient to breathe not over three times. It is a powerful heart stimulant.

I can not leave this subject with this hurried and cursory con-consideration without expressing my hope that the better practitioners will give it more attention than they have heretofore, not only that our patients may have the benefit and come to us with less of dread, but that the whole subjects may be taken out of the hands of the demoralizing element in our profession. Where wise men fear to venture, quacks rush in and claim the victory. If we are ever to overcome unprofessional practice, we must disarm those who engage in it. There is no fortress behind which they are to-day so solidly intrenched as this subject which is before us to-night. Is it not our duty to the younger men in the profession who are called upon to meet these unprofessional business methods and practices, to arm them with definite ammunition of equal, if not superior quality?

